# The Response of Cerebral Cortex to Haemorrhagic Damage: Experimental Evidence from a Penetrating Injury Model

**DOI:** 10.1371/journal.pone.0059740

**Published:** 2013-03-21

**Authors:** Sivaraman Purushothuman, Lauren Marotte, Sally Stowe, Daniel M. Johnstone, Jonathan Stone

**Affiliations:** 1 Bosch Institute and Discipline of Physiology, University of Sydney, Sydney, NSW, Australia; 2 Division of Biomedical Sciences and Biochemistry, Research School of Biology, Australian National University, Canberra, ACT, Australia; Univ. Kentucky, United States of America

## Abstract

Understanding the response of the brain to haemorrhagic damage is important in haemorrhagic stroke and increasingly in the understanding the cerebral degeneration and dementia that follow head trauma and head-impact sports. In addition, there is growing evidence that haemorrhage from small cerebral vessels is important in the pathogenesis of age-related dementia (Alzheimer’s disease). In a penetration injury model of rat cerebral cortex, we have examined the neuropathology induced by a needlestick injury, with emphasis on features prominent in the ageing and dementing human brain, particularly plaque-like depositions and the expression of related proteins. Needlestick lesions were made in neo- and hippocampal cortex in Sprague Dawley rats aged 3–5 months. Brains were examined after 1–30 d survival, for haemorrhage, for the expression of hyperphosphorylated tau, Aβ, amyloid precursor protein (APP), for gliosis and for neuronal death. Temporal cortex from humans diagnosed with Alzheimer’s disease was examined with the same techniques. Needlestick injury induced long-lasting changes–haem deposition, cell death, plaque-like deposits and glial invasion–along the needle track. Around the track, the lesion induced more transient changes, particularly upregulation of Aβ, APP and hyperphosporylated tau in neurons and astrocytes. Reactions were similar in hippocampus and neocortex, except that neuronal death was more widespread in the hippocampus. In summary, experimental haemorrhagic injury to rat cerebral cortex induced both permanent and transient changes. The more permanent changes reproduced features of human senile plaques, including the formation of extracellular deposits in which haem and Aβ-related proteins co-localised, neuronal loss and gliosis. The transient changes, observed in tissue around the direct lesion, included the upregulation of Aβ, APP and hyperphosphorylated tau, not associated with cell death. The findings support the possibility that haemorrhagic damage to the brain can lead to plaque-like pathology.

## Introduction

Recent advances in cerebral imaging have provided evidence that haemorrhage occurs in the brain not only in clinically evident haemorrhagic stroke, but also in clinically ‘silent’ microbleeds or ‘silent strokes’ which, like stroke, occur with increasing frequency with age [1], and contribute to the dementia of the aged [2], [3]. Neuropathological studies [4], [5], [6], [7], [8] have provided evidence that haemorrhage from or dysfunction of cerebral capillaries may be important in the formation of the plaques characteristic of age-related dementia (Alzheimer’s disease) [9], [10], [11], and risk-factor analyses indicate that age-related dementias are strongly vascular. Small, clinically silent haemorrhages may be a major factor in the burden of dementia which afflicts the population in an age-related pattern.

The present study examines the neuropathology induced in cerebral cortex of the young, healthy rat by a thin needle penetrating neocortex and hippocampus. The response of the cortex to the injury has been examined with immunohistochemistry, using techniques which show neuronal death, loss of synapses, microglial and macroglial proliferation, and proteins and peptides (APP, Aβ, tau) whose expression is considered important in Alzheimer’s disease. This allows a comparison with ageing human brain, and a test of the idea that plaques form at the sites of haemorrhage from cerebral capillaries [4], [5], [8].

The idea that plaques form where capillaries fail has been formulated in several ways in prior studies - that plaques form when capillaries cease to pass blood due to age-related defects in their structure, creating local hypoxia [12], [13], [14], [15]; or that plaques form where capillaries lose barrier properties and release proteins or other substances into the neuropil [7], [16]. The present study tests whether haemorrhagic injury is sufficient to generate features of senile plaques, and also provides evidence that the area of direct injury and haemorrhage, and the flanking regions, respond in distinctive ways, which contribute differently to the end-stage pathology observed.

## Materials and Methods

### Ethics Statement

All research on banked, deidentified human brain tissue was approved by the University of Sydney Human Research Ethics Committee. All animal-based research was approved by the University of Sydney Animal Ethics Committee and was conducted in accord with guidelines of the National Health and Medical Research Council of Australia.

### Experimental Material from Rats

Sprague Dawley (SD) albino rats of both genders, 3–5 months old and weighing approximately 300–400 g, were caged in pairs and kept in a 12 h light (<5 lux)/dark cycle at 22°C. Food pellets and water were available *ad libitum*.

#### Surgical procedure

**Figure 1:**
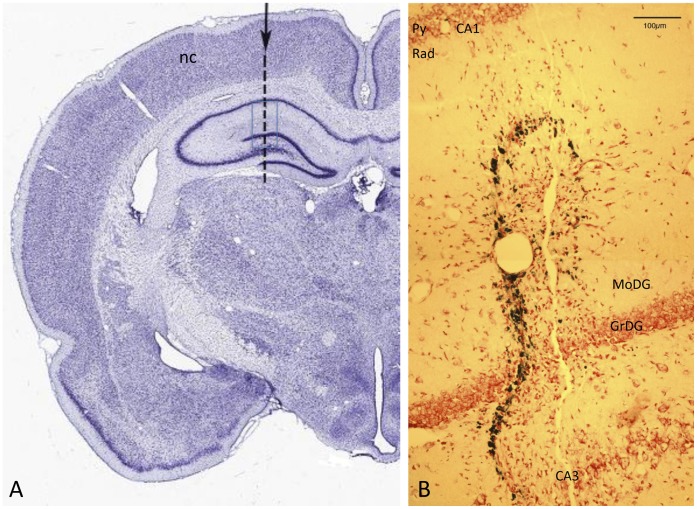
The needlestick lesion. A: Coronal section of rat brain, ∼3 mm anterior to bregma, stained with Thionine [74]. The arrow and dotted line show the targeted position of the needle track. The track traverses neocortex (nc) and hippocampus, from CA1 across the dentate gyrus and CA3. The blue rectangle shows the area imaged at higher power, from an experimental animal, in B. B: Needle track crossing layers of the hippocampus, in the region marked by the rectangle in A. The track is evident as a string of Prussian-blue labelled deposits, close to a linear break in the neuropil. Neutral red counterstain. *Py* – pyramidal layer of CA1; *Rad* - stratum radiatum of CA1; *MoDG* – molecular layer of the dentate gyrus; *GrDG* – granular layer of the dentate gyrus.

Rats were anaesthetised with isoflurane (IsoFlo, Abbott Laboratories, U.S.A) and were given a systemic analgesic (Rimadyl, Pfizer, 5 mg/kg subcutaneous). The scalp was shaved and incised along the midline to expose the dorsum of the skull. The head was placed in a stereotaxic frame and a small hole (1.35 mm in diameter, located 3.5 mm caudal to the bregma landmark and 2 mm from the midline) was drilled through the skull in each side, leaving the dura mater intact. A sterile, thin (0.64 mm diameter) needle was inserted vertically to a depth of 4.5 mm from the dura, into the cerebral cortex in each hemisphere. This approach was taken, following [17], so that the needle would penetrate both the neocortex and the hippocampus (Figure 1A). The needle was held in place for 5 seconds, and then removed. After completing the needle insertions, the incision was infiltrated with a local anaesthetic (2% Lignocaine, Troy Laboratories, Australia) and sutured.

#### Brain collection, fixation, embedding and sectioning

Brains were studied from 35 animals, comprising 5 animals at 1 d survival post-lesion, 5 at 3 d, 2 at 5 d, 3 at 7 d, 4 at 14 d, 1 at 19 d and 6 at 30 d, 5 age-matched controls and 4 aged (>12 months) controls. Rats were euthanised by intraperitoneal injection of sodium pentabarbitone (60 mg/kg).

Brains collected for immunoblotting (1 at each time point) were not fixed. Hemi-coronal 2 mm thick slices from the region including the lesion were made with an ice-cold rat brain slicer block (BSRAS001-1, Zivic Instruments, U.S.A) and immediately placed in chilled microcentrifuge tubes, snap-frozen in liquid nitrogen and stored at −80°C.

Brains for histology and immunohistochemistry were fixed by perfusion. Briefly, 0.01% heparin sodium salt and 1% sodium nitrite (Sigma) were injected into the left ventricle. The rats were then perfused with 0.1 M saline (2 min) followed by 4% paraformaldehyde in 0.1 M phosphate-buffered saline (PBS) for 10 min. Brains were removed and further fixed by immersion in 4% paraformaldehyde for 3 h. Brains were cut mid-sagittally and hemispheres cryoprotected in 15% sucrose in 0.1 M PBS overnight. Hemispheres were embedded in TissueTek OCT compound (ProSciTech) and cryosectioned at 12 µm in the coronal plane.

#### Antibodies used

The antibodies used are listed in Table 1. Primary antibodies raised in mouse are monoclonal, while those raised in rabbit are polyclonal.

**Table 1:** Antibodies used for Western Blotting (WB) and immunohistochemistry (IHC).

	Antigen/name	Epitope/Clone	Host species	Working dilution IHC/(WB)	Supplier & Catalogue no.
Primary Antibodies	Alzheimer precursor protein	N-terminal pre-A4/22C11	Mouse	1∶200 (2500)	Millipore MAB348
	β-Amyloid	Aβ_1–16_/6E10	Mouse	1∶500 (1000)	Covance SIG39320
	β-Amyloid	Aβ_17–24_/4G8	Mouse	1∶500 (1000)	Covance SIG39220
	Aβ oligomers	Prefibrillar oligomers/A11	Rabbit	1∶1350 (500)	Millipore AB9234
	CD11b	OX-42	Mouse	1∶50	Millipore CBL1512
	GFAP	−	Rabbit	1∶1000	Dako Z0334
	Neuronal Nuclei (NeuN)	A60	Mouse	1∶100	Millipore MAB377
	Synaptophysin	p38	Rabbit	1∶200 (10000)	Dako A0010
	Phosphorylated Tau	AT8	Mouse	1∶500	Innogenetics 90206
	GAPDH	−	Mouse/Rabbit	(1∶3000)	Sigma G8795/9545
Secondary Antibodies	Alexa Fluor 488-conjugated	Mouse IgG	Goat	1∶1000	Molecular Probes A11029
	Alexa Fluor 594-conjugated	Rabbit IgG	Goat	1∶1000	Molecular Probes A11037
	Biotinylated	Mouse IgG	Goat	1∶200	Vector BA9200
	HRP-conjugated	Mouse/Rabbit IgG	Goat	(1∶3000)	Millipore AP308/307

Working concentrations for Western Blotting are indicated in brackets.

#### Western blotting

Tissue slices were homogenised in radio-immunoprecipitation assay (RIPA) buffer (Millipore) containing PhosStop (Roche Diagnostics), and 1∶100 protease inhibitor cocktail (Sigma). The homogenate was centrifuged at 12,000×g for 10 min at 4°C and the supernatant retained.

Protein concentration was determined using the Pierce BCA Protein Assay kit (Thermo Scientific). Protein samples (50 µg per lane) and 0.09 ng/µl of Aβ 1–40 protein standards (Bachem) were resolved by SDS-PAGE under reducing conditions, using either 10% Tris-Glycine gels or 10–20% Tris-Tricine gels (Biorad) for larger (70–100 kDa) proteins and smaller peptides, respectively. Tris-Glycine gels were blotted to a 0.45 µm PVDF membrane and Tris-Tricine gels were blotted to a 0.2 µm nitrocellulose membrane. Membranes were blocked in 5% nonfat dry milk in TBS with 0.1% Tween 20 (TBST) for 1 h. Membranes were incubated overnight at 4°C with primary antibodies (APP, A11, 6E10, 4G8– Table 1) followed by 1∶3000 HRP-conjugated mouse or rabbit secondary antibody (Millipore), as appropriate. Blots were visualised by chemiluminescence detection with an HRP substrate (Millipore). Blots were stripped with Reblot Plus solution (Millipore) and probed for glyceraldehyde 3-phosphate dehydrogenase (GAPDH), to establish internal loading controls. When probing for Aβ monomers and low molecular weight (<25 kDa) oligomers, nitrocellulose membranes were boiled for 5 min in PBS immediately after transfer and prior to blocking. A signal boost (Merck) was used as a diluent for 6E10 and 4G8 antibodies on the nitrocellulose membranes. Band intensities were measured with Image J 1.43, and normalised to GAPDH.

#### Immunohistochemistry

**Single immunohistochemistry** using fluorescent labelling was performed to demonstrate the antigens Aβ, APP, oligomeric Aβ hyperphosphorylated tau, using the antibodies 4G8, 6E10, anti-APP, A11 and AT8. Neurones and microglia were labelled using monoclonal antibodies against the markers NeuN (1∶100) and CD11b (1∶50), respectively. Antigen retrieval was performed using 90% formic acid (pH 1.6) for 10 min for 4G8, 6E10, and APP antibodies or 80% Reveal It (ImmunoSolution) in dH_2_O at 37°C for 20 min for the other antibodies. Sections were blocked in 10% normal goat serum (Sigma) in PBS and 0.3% Triton-X 100 (Sigma) before overnight incubation at 4°C with primary antibodies (Table 1) diluted in 1% NGS/PBS. Sections were then incubated with 1∶1000 goat anti-mouse Alexa Fluor 488 secondary antibody (Molecular Probes) for 3 h at room temperature or 80 min at 37°C. Sections were counterstained for nuclear DNA using 1∶10,000 bisbenzimide for 2 min (Hoechst).

Single immunohistochemistry using 3,3′ diaminobenzidine (DAB) as the chromogen was performed with the antibodies anti-APP, 6E10, 4G8, to confirm the observations using immunofluorescence, as well as the anti-tau antibody AT8. Peroxidase activity in the sections was quenched with 3% H_2_O_2_ in 50% methanol/PBS for 15 min. The sections were then washed and those to be stained for AT8 underwent antigen retrieval using 0.1 M sodium citrate buffer (75°C for 20 min, then allowed to cool at room temperature for 45 min). For other antigens, the sections were processed with formic acid as described above. After incubating overnight at room temperature with the relevant primary antibodies, sections were washed in PBS and were incubated in biotinylated anti-mouse IgG for 1 h followed by extravidin peroxidase (1∶1000; Sigma E2886) for 2.5 h. The sections were then washed and developed with DAB (0.5 mg/ml, 3–5 min in 0.003% hydrogen peroxide) as the chromogen.

**Double immunohistochemistry** was used to show the co-occurrence of monomeric Aβ, oligomeric Aβ or APP and a second protein, either the intermediate filament GFAP, a marker of astrocytes, or synaptophysin, found in synaptic vesicles. Sections were treated in 90% formic acid (pH 1.6) for 10 min for antigen retrieval before blocking in 10% NGS. Sections were incubated overnight at room temperature with a mixture of one mouse monoclonal antibody (to APP or Aβ) and one rabbit polyclonal antibody (to GFAP, synaptophysin or oligomeric Aβ), diluted in 1% NGS/PBS. Sections were then incubated in a mixture of Alexa Fluor-488- and Alexa Fluor-594-tagged secondary antibodies specific to the primary antibodies’ host species. Following secondary antibody treatment, sections were counterstained with bisbenzimide. Fluorescent images were collected using a Zeiss deconvolution system, and were analysed using ImageJ software (National Institute of Health, USA).

**Negative controls** were routinely prepared by following all the above steps, but omitting the primary antibodies.

#### TUNEL staining

To detect apoptotic cells, sections were labelled with the TUNEL technique [18] to identify fragmentation of DNA characteristic of dying cells, following published protocols [19]. Briefly, sections were washed in terminal deoxynucleotidyl transferase (TdT; Roche) buffer for 10 min at room temperature and then in a reaction mix of TdT (0.03 enzyme unit/µl) and biotinylated deoxyuridine triphosphate (dUTP; 4 enzyme unit/µl; Roche) for 60 min at 37°C. The sections were then incubated with a stop reaction containing 300 mM NaCl and 30 mM sodium citrate for 15 min at room temperature. Sections were blocked for nonspecific binding in 10% NGS for 15 min and then incubated for 30 min at 37°C with streptavidin-conjugated Alexa Fluor 594 (1∶1000), followed by counterstaining with bisbenzimide. For some sections, the TUNEL reaction was followed by single immunohistochemistry, as described above, with Alexa 488 as the reporting chromophore.

Counts of TUNEL^+^ profiles were made at 20× magnification along the needle track in both the hippocampus and the neocortex, from at least five sections from each brain hemisphere. The counts were averaged and expressed in TUNEL^+^ profiles/mm along the needle track.

#### Staining for iron

The **Prussian blue** (Perls) **reaction** was used to identify iron deposits in the brain, to detect sites of haemorrhage. Frozen sections were rinsed in 70% ethanol and placed in a solution comprising equal portions of 4% HCl and 4% potassium ferrocyanide for 30 min at room temperature. They were then washed in distilled water for 5 min, and incubated in 1% neutral red solution for 3 min, as a nuclear counterstain.

#### Congo red and thioflavin staining

Selected sections labelled with the Perls reaction were second-labelled with either 1% Thioflavin S (Sigma) for 5 min (following [20]) or with Congo Red (Sigma, Putchler’s modification [21], [22]). Briefly, sections were treated with 2.9 M sodium chloride in 0.01 M NaOH for 20 min and staining with filtered 0.2% Congo red in alkaline sodium chloride solution for 1 h.

### Human Material

#### Source

Human postmortem tissues from cases with a diagnosis of Alzheimer’s disease, without a clinical history of stroke, were obtained from the Sydney Brain Bank. The tissue comprised cerebral cortex from the temporal and parietal lobes, from 4 cases aged 69–100 years, at stages V and VI of the diseases [23]. The material had been fixed and stored in 4% formalin.

#### Cryosectioning and immunohistochemistry

Blocks of cortical tissue were cryosectioned coronally at 40 µm and free-floating sections collected. Using the protocols described above, sections were immunolabelled for Aβ and its precursors (4G8 and 6E10 antibodies), for oligomerised Aβ (A11 antibody), for APP, for the astrocyte-specific intermediate filament protein GFAP, and for tau.

#### Prussian blue labelling combined with Thioflavin S or congo red

Labelling for haem-derived iron was carried out as described above, with two forms of enhancement. Briefly, some free-floating sections were first incubated in 4% HCl and 4% potassium ferrocyanide for 30 min, followed by enhancement with diaminobenzidine (0.67 mg/ml in TBS) and hydrogen peroxide (final dilution 0.001%) for 5 min. These sections were second-labelled with either Thioflavin S or with Congo Red, as described above. Other sections were incubated for a much longer period (56 h) in the same HCl and ferrocyanide solution, following [4], and then counterstained in 1% Neutral Red solution for 5 sec.

#### Thin (0.2 µm) sections and immunohistochemistry

A variant of the “array tomography” technique of Micheva and Smith [24] was used with immunolabelling on adjacent thin sections, imaging to maximise the information obtained from autofluorescence. Blocks (2 mm cubed) were cut from the parietal neocortex (84 years, Stage V–VI) and embedded in resin. Serial sections were cut at 200 nm using a diamond knife (Histoknife, Diatome, AG Biel Switzerland) and collected on water droplets on 10-cell Teflon-coated slides (ProSciTech, Australia), one section per drop. Slides were dried at 60°C and stored at 4°C.

The series used here extended for 250 sections (50 µm). Sections were collected separately and stained individually. To minimise ambiguities arising from co-localisation and autofluorescence, only single-label immunohistochemistry was performed on this material. Sections were blocked for 30–60 min in 5% NGS before incubation (3–4 h at room temperature or overnight at 4°C) in primary antibodies diluted in 5% NGS (usually 1∶100). Sections were then incubated in AlexaFluor secondary antibodies (1∶2000) for 1 h at room temperature, followed by counterstaining with bisbenzimide.

We also assessed autofluorescence, which is rich in ageing brain tissue. Yellow- or reddish-brown autofluorescence is exhibited by lipofuscin/lipopigment [25] and/or haemosiderin [26] granules, while blue or violet fluorescence is exhibited by senile plaques [27], [28], elicited by UV or violet (405 nm) illumination and believed to stem from Aβ peptide or a closely associated plaque constituent.

#### Imaging

To construct montages, sections were viewed using a Cytoviva (Cytoviva Inc., Auburn, AL, USA) dark field condenser system with Dual Mode Fluorescence module on an Olympus BX31 microscope with a 40X NA 0.75 UPlan FLN Olympus objective and an Exfo X-Cite 120 light source. The emission filter was a 3 colour multiband (440–470, 520–545, 610–645 nm) filter, and the excitation filter was also a 3 colour multiband (390–410, 480–510,560–590), except for a very few cases in which the Alexa 488 signal was relatively weak, when a single band 480–510 excitation filter was used with the same emission filter. Images (24 Mb, 2048×2048 pixel, 14 bit) were taken with a SPOT Flex Mosaic colour camera (Diagnostic Instruments, MI, USA), operated in “4-shot” mode with a total exposure time of 20 sec and a gain of 1. Approximately 50 overlapping images were taken for each section. In Adobe Photoshop, the images were treated with a high pass filter and the intensity histogram adjusted before overlaying manually to produce a single montage for each section.

## Results

The target needle track, shown in Figure 1A on a coronal section of the rat brain (corresponding to ∼3.8 mm posterior to bregma [17]), traverses neocortex and hippocampal cortex, including the junction of the dentate and hippocampal gyri. Figure 1B shows an area equivalent to that boxed in Figure 1A from one experimental brain, with the needle track crossing the granule layer of the dentate gyrus. The dark labelling is the Perls/Prussian blue reaction for iron, showing that the residue of bleeding induced by the needle was closely restricted to the track. Also evident with this labelling is a physical split in the tissue; overall the pathology induced by the needle seems confined to within 100 µm either side of the track.

Tested with other labels (below), the pathology along the needle track was dominated by long-lasting features - haem deposition, neuronal death, gliosis and the formation of plaque-like extracellular deposits of autofluorescent material. In the flanking regions, the reaction of the tissue was transient and reversible; we observed the transient intracellular upregulation of Aβ and/or APP (labelled with the 4G8 or 6E10 antibodies), of Aβ oligomers and of hyperphosphorylated tau. The 4G8 and 6E10 labelling was prominent, in neurones and astrocytes, confirming earlier studies [29]; in our material, labelling with these two antibodies was essentially identical. Cell death was not detected in the flanking regions, except where the needle passed through the pyramidal layer of the hippocampus.

### Along the Track

**Figure 2:**
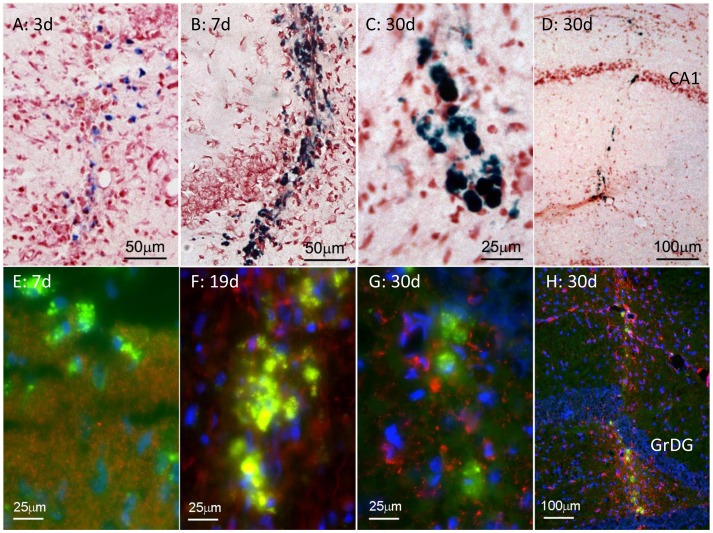
Formation of haem deposits and fluorescent deposits after needle stick lesion. A–D: Prussian blue-labelled (haem) deposits, 3–30 d after lesion. Deposits formed along the needle track, and were tightly confined to the track. They were relatively diffuse at 3 d, and became dense by 7 d. E–H: Fluorescent deposits along the needletrack 7–30 d after the lesion. Like haem deposits, the fluorescent deposits were distributed along, and confined to the needle track (H). The red label shows synaptophysin labelling in E, and astrocytes (GFAP) in F–H. The GFAP labelling is tightly confined to the needletrack in H. The blue fluorescence is bisbenzimide labelling of nuclear DNA. The needle track in H is through the hippocampus; GrDG indicates the granule layer of the dentate gyrus.

The Perls (Prussian Blue) reaction labels iron moieties released into the tissue by the injury, presumably as a result of haemorrhage. This labelling appeared relatively diffuse at day 1 (not shown). By day 3, presumably as a result of the action of phagocytotic micro- and macroglia (below), the blue deposits were more granular and compact (Figure 2A). By day 7, they appeared dark blue and condensed (Figure 2B), and they retained this appearance at the longest survival examined (30 d, Figures 2C, D). Spatially, they were confined closely to the needle track (Figure 1B).

**Figure 3:**
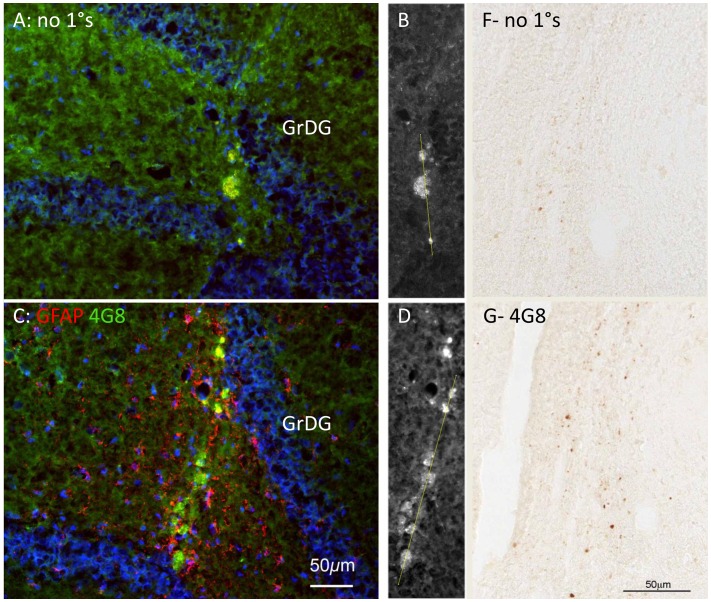
Analysis of antibody-specific fluorescence vs. autofluorescence. A, C: Neighbouring sections through a needle track 30 days after the lesion. In A, no primary antibodies were used; these are negative controls. In C, antibodies for GFAP (red) and APP/Aβ (green) were used. Exposure times, excitation intensities and all other settings were held constant between A and C. Scale bar in C also applies to A, B and D. Blue fluorescence is bisbenzimide staining of nuclear DNA. B, D: The green fluorescence in A and C is shown in white. Sample lines used for the analysis in Figure 4 are shown, traversing the fluorescent bodies. F, G: Neighbouring sections through a needle track 30 d after the lesion, showing endogenous peroxidase activity in negative control material; and specific labelling with the 4G8 antibody (which labels APP and Aβ) along the needle track. Scale bar in G also refers to F.

Extracellular deposits (ECDs) formed along the needle track; they were first detected at day 7 (Figure 2E) and persisted until day 30 (Figures 2F–H). They were similar in size to the Prussian blue-labelled deposits (<50 µm in diameter) and to human senile plaques (below). In material labelled with the 4G8 or 6E10 antibodies (which label APP and Aβ), these ECDs fluoresced brightly. Their fluorescence may arise from autofluorescence of lipofuscins that have accumulated subsequent to the haemorrhage and tissue destruction caused by the injury, and from specific labelling with 4G8 or 6E10. To test this point, the following analysis was undertaken. We labelled a section showing ECDs with 4G8, with an antibody to GFAP and with bisbenzimide, which labels DNA (to demonstrate cell nuclei) (Figure 3C). A neighbouring section, labelled by the same protocol but omitting the primary antibodies, served as a negative control (Figure 3A). Using Image J we sampled fluorescence intensities along sampling lines crossing ECDs in the negative and positive samples; the sampling lines used are shown in Figures 3B and D. Immunohistochemistry with the 4G8 antibody using a non-fluorescent marker (Figure 3F, G) also showed evidence of specific labelling of Aβ and/or APP deposits along the needle track. The dark labelling in G, compared to the negative control material in F, again suggests deposits distributed along, but limited to the track.

**Figure 4:**
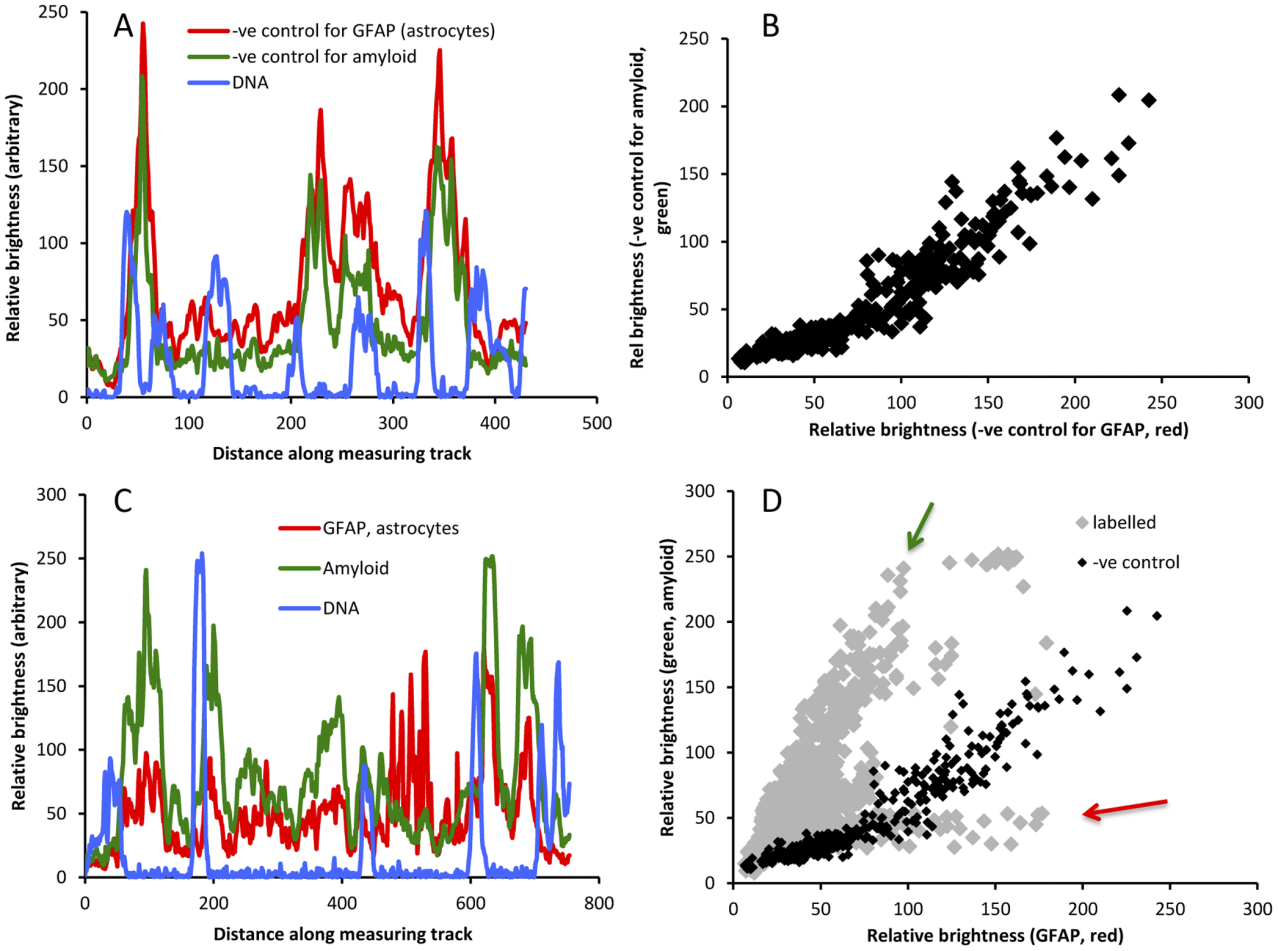
Analysis of antibody-specific fluorescence vs. autofluoresence. A, B: A shows the variation of red, green and blue fluorescence in negative control sections (along the line shown in Figure 3B). The blue fluorescence is bisbenzimide labelling of nuclear DNA; the red and green fluorescence is autofluorescence. B shows that red and green fluorescence levels co-vary. C, D: C shows the variation of red, green and blue fluorescence in the section in Figure 3C, along the line shown in Figure 3D. This material was labelled for GFAP (red), amyloid (green) and nuclear DNA (using bisbenzimide). D shows the red vs green plot for points along the line in Figure 3D. The dark points reproduce the co-variance seen for negative control material in B. The red and green arrows indicate points that show high red fluorescence (GFAP) and high green fluorescence (amyloid).

The intensity profiles obtained along the tracks shown in Figure 3B,D were analysed in two ways. When intensity was plotted against distance, it can be seen that in the negative control material, red and green signals largely co-varied (Figures 4A, B), while the blue signal showed independent changes. This suggests that the ECDs emitted at similar intensities in the red and green ranges (suggesting autofluorescence), while the blue emissions represented the positive labelling of DNA (cell nuclei) with bisbenzimide. In the positive labelled material, by contrast, the fluorescence/distance graph (Figure 4C) shows independent green and red peaks, and the green vs red plot (Figure 4D, grey symbols) is distinct from Figure 4C. Many sampling points showed high red values and low green values (red arrow), and many points showed high green values and low red values (green arrow). The former are from the labelling of GFAP in astrocytes; the latter (we argue) are from 4G8 labelling of ECDs. One negative result should be noted. Congo Red and Thioflavin S did not label the Aβ^+^ deposits in rat brain, in contrast with human senile plaques (below).

**Figure 5:**
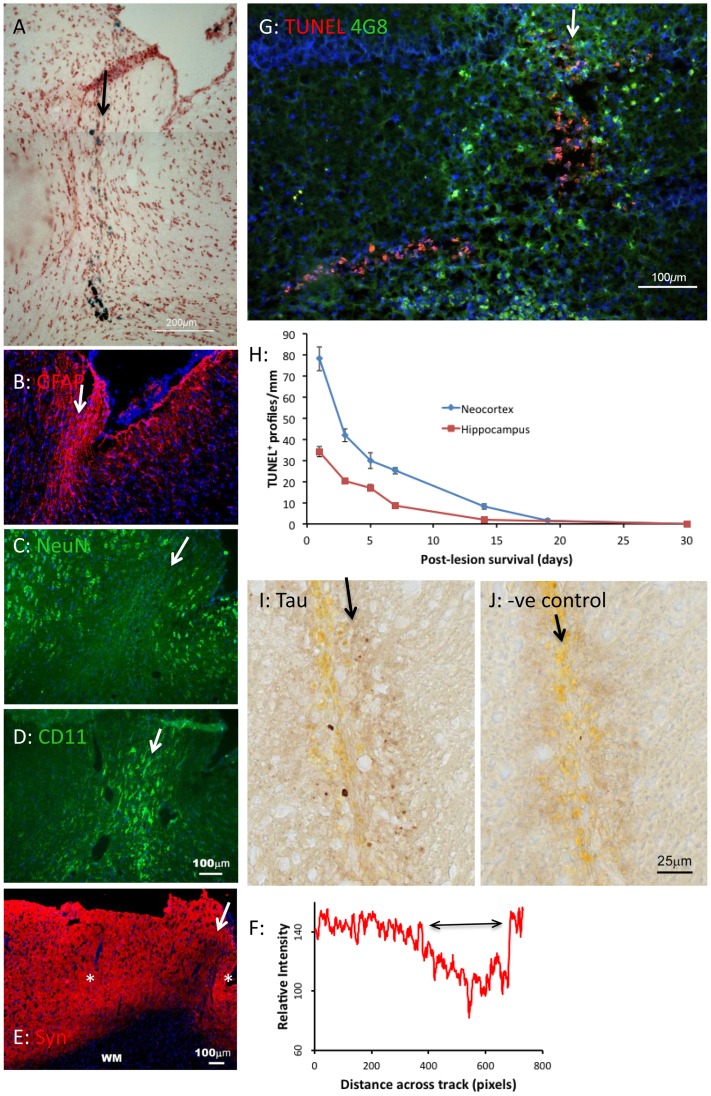
Neuropathology of needlestick lesion. The arrows indicate the locus and direction of the needle track. The blue labelling in B to E and G is bisbenzimide labelling of nuclei. A–E: Neighbouring sections of neocortex 30 d after the lesion showing haem deposition (A, Perls reaction on a Neutral Red counterstained background); astrogliosis (B, GFAP labelling) along the track and spreading beyond it; the loss of nerve cells along the track (C, NeuN labelling); microgliosis along the track (D, labelling with CD11); loss of synaptophysin labelling along the track (E); quantification of synaptophysin labelling (F) along the line in E between the asterisk. G: Most cell death (labelled red with the TUNEL technique) was concentrated along the needle track (arrows), whether in neocortex or hippocampus. This image shows the only exception observed - TUNEL labelling shown here in the granule layer of the dentate gyrus, extending laterally (to the left) from the track. The 4G8 antibody has also been applied (green). H: Quantification of TUNEL^+^ (dying) cell nuclei along needle tracks through neocortex and hippocampus, as a function of survival time (5 sections counted per time point, error bars showing standard errors of the means). I, J: AT8 labelling of hyperphosphorylated tau in extracellular deposits along a needle track in neocortex, with DAB as the reporting chromagen (J is the negative control for I).

Labelling with the AT8 antibody gave evidence that the extracellular deposits that form along the track also contain hyperphosphoryated tau (Figure 5I, J). Like haem^+^ and Aβ^+^ deposits, the AT8^+^ deposits also appeared first at 7 d, and remained prominent at 30 d, suggesting that some deposits could contain all three antigens.

The death of neurones along the needle track was assessed in 3 ways. TUNEL labelling showed dying (fragmenting DNA labelled red) cells along the needle track (arrows in Figure 5G). When labelling was quantified, as the number of TUNEL^+^ profiles per millimetre of track, cell death appeared greatest at 1 d survival (the earliest time point examined), the number of TUNEL^+^ (dying) cells then falling steadily, reaching background levels by day 19 (Figure 5H). In addition, labelling with an antibody to synaptophysin (Figure E,F) and with the antibody NeuN (specific for the nuclei of neurones, Figure 5C) shows that synaptic labelling was decreased along the needle track (between the asterisks in Figure 5E, across the double-headed arrow in Figure 5F), and that neuronal cell bodies were absent from the site of the track (Figure 5C). That is, neurones and some of the synapses they form appeared to be destroyed by the lesion.

Figure 5G was chosen to show that in the hippocampus, cell death (red TUNEL^+^ nuclei) was observed to extend beyond the site of the track, in the granule cell layer of the dentate gyrus (towards the bottom left in Figure 5G), but not in other layers of the hippocampal cortex, or in the neocortex. This was observed in 2 brains, 3 and 5 d post-lesion.

Despite the severe loss of neurones along the track, the site appeared cellular, when viewed with a DNA label, such as the nuclear fast red labelling in Figure 5A, suggesting that the site was re-populated. The cells that repopulate the neurone-depleted site include microglia (shown with the CD11b antibody in Figure 5D) and astrocytes (Figure 5B). This repopulation was evident by 3 d post-lesion; glia became a prominent and stable feature of the lesion by 7 d.

**Figure 6:**
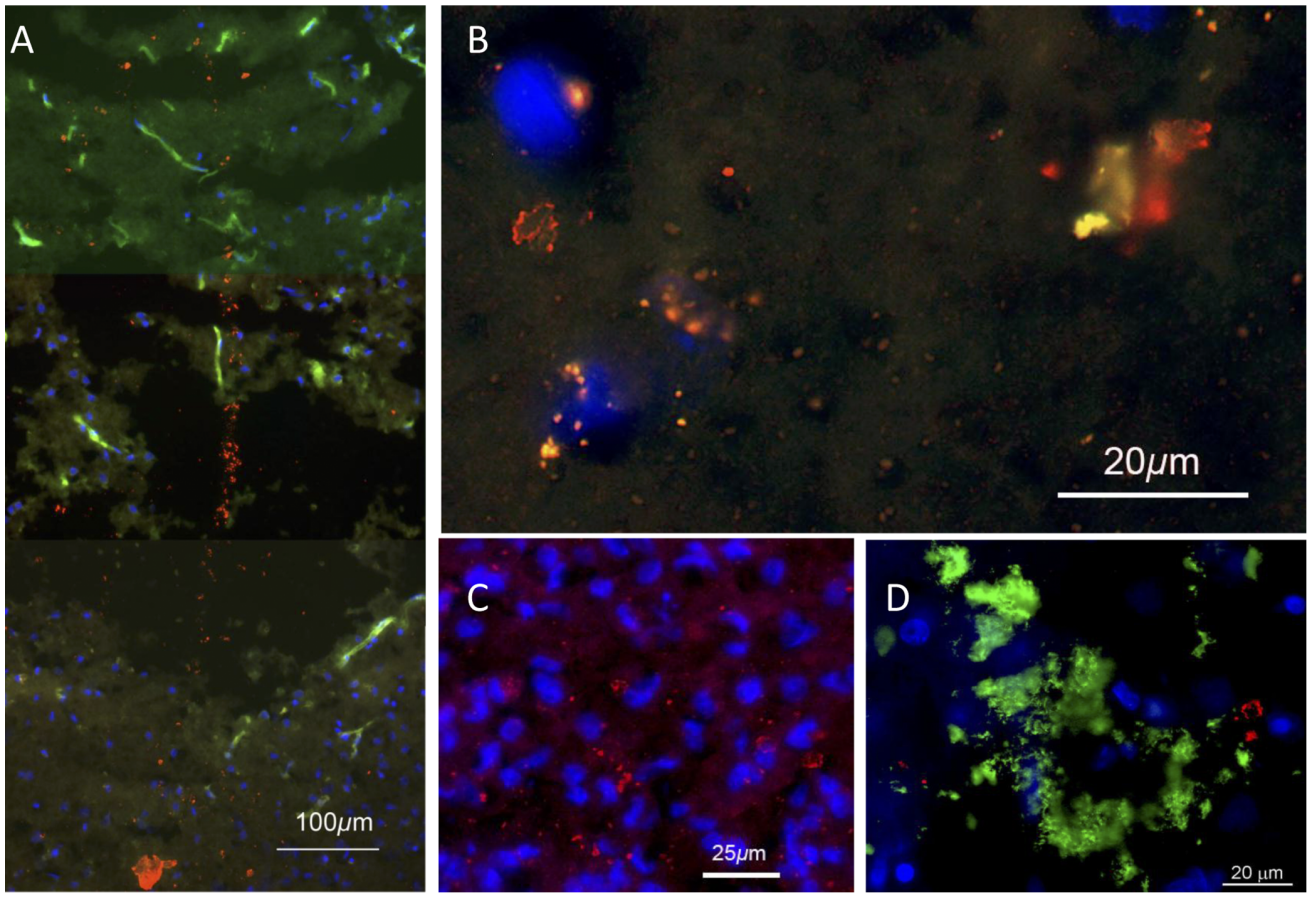
Immunohistochemical localisation of oligomeric Aβ, 3–5 d post-lesion (A,B,C) and near a human plaque (D). Blue fluorescence is bisbenzimide labelling of nuclear DNA. A: In favorable sections A11 labelling (red) could be seen along the needle track. The green fluorescence is autofluorescence, most marked in blood vessels. B, C: Further from the track, A11 labelling was often punctate, but small crystal-like structures were also labelled, as previously reported [36], [37]. D: A11 labelling in human neocortex was also often punctate, but larger crystalline structures were seen, usually away from or (as here) at the edge of a plaque. The green label is 4G8 (Aβ and precursors).

Oligomerisation of Aβ is considered to be a step to the formation of insoluble deposits of amyloid [30] and many studies [31], [32], [33] have reported that the soluble, oligomerised forms are neurotoxic. In our material, at 1–3 d after the needlestick, oligomerised Aβ was detected with the A11 antibody along the needle track (Figure 6A). It did not appear to concentrate in ECDs.

### Along the Flanks of the Track

Previous studies have noted that focal lesions to brain tissue induce transient upregulation of APP and Aβ at the margins of the directly affected tissue [34] and upregulation of hyperphosphorylated tau has been reported within 24 h of traumatic brain injury in humans [35]. We observed similar changes in tissue flanking the needle track.

**Figure 7:**
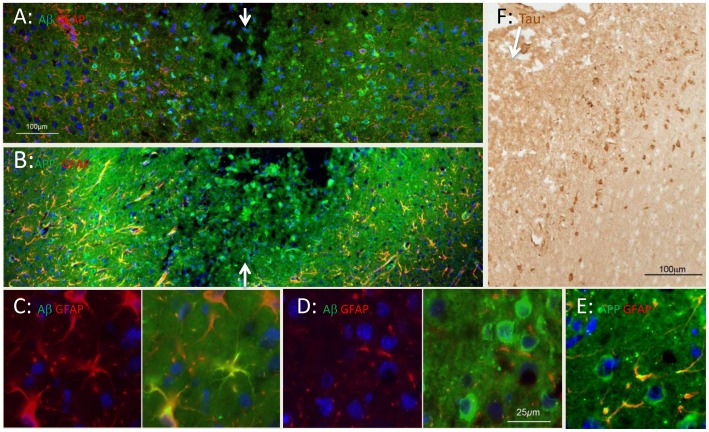
Immunohistochemical localisation of tau, APP, Aβ and precursors, at 3 d post-lesion. A: The needle track is marked by arrows. On each side of the track, 4G8 (green) labelling (of Aβ and its precursors, including APP) is upregulated in the somas of neurones and astrocytes, for up to 200 µm. The red labelling is GFAP (astrocytes). B: In an adjacent section, APP labelling (green) is also upregulated along the flanks of the track. Labelling with the APP antibody appears more extensive than with the 4G8 antibody, suggesting that 4G8 shows some selectivity for Aβ. C: Left and right panels show labelling for GFAP and for both GFAP and 4G8, at higher power. The GFAP^+^ cells (astrocytes) are also 4G8^+^. D: Left and right panels show labelling for GFAP and for both GFAP and 4G8, at higher power. Neuronal somas (larger, rounded, GFAP^−^) are strongly 4G8^+^ (green). E: APP labelling (green) is found in neuronal somas and co-localises with GFAP (red) in astrocyte processes. F: Immunolabelling for hyperphosphorylated tau, at the edge of a needle track in neocortex. The arrow marks the needle track; labelling with DAB. The scale in A applies to B; the scale in D applies also to C and E. Blue is nuclear DNA labelled with bisbenzimide.

The most striking feature of the regions flanking the needle track was the prompt increase of 4G8 or 6E10 labelling in cells on the flanks of the track (Figure 7A); as Table 2 shows, this upregulation was transient, limited to <7 d. APP was also expressed promptly and transiently in these flanking regions (Figure 7B, Table 2). Using a non-fluorescent marker (Figure 7F) we obtained evidence of upregulation of hyperphosporylated tau intracellularly, in neurones flanking the needletrack. This upregulation was also transient, limited to <7 d (data not shown).

**Table 2:** The time course of neuropathological events induced by needlestick injury.

Post lesion time	Area of brain	Aβ- or tau- related					Gliosis		Loss of neurones (along track)			Haemorrhage	Extracellular deposits
		Intracellular expression in neurones/neuroglia in flanks											
		APP	Aβ	Oligomeric Aβ	Hyper-phosph tau	Oligomeric Aβ (track)	Astro-cytes (GFAP)	Microglia (CD11b)	Synaptic loss (synapto-physin)	Neuron loss (NeuN)	TUNEL-labelled cells/mm	Perls reaction	Haem^+^, 4G8^+^, AT8^+^
1 d	Neocortex	+++	+++	−	+++	+++	−	+	−	−	78	+	−
1 d	H’campus	+++	+++	−	+++	+++	−	++	−	−	28	+	−
3 d	Neocortex	++	++	++	++	++	+	+	−	−	42	+	−
3 d	H’campus	+	+	++	++	+	+	++	−	−	20	++	−
5 d	Neocortex	−	+	+++	+	+	+	++	−	−	42	+	−
5 d	H’campus	−	+	+	−	−	+	++	−	−	34	++	−
7 d	Neocortex	−	+	+	−	−	+	++	−	−	27	++	+
7 d	H’campus	−	+	−	−	−	+	++	+	+	9	++	+
14 d	Neocortex	−	−	−	−	−	++	++	++	++	8	++	++
14 d	H’campus	−	−	−	−	−	+++	+++	++	+++	2	+++	++
19 d	Neocortex	−	−	−	−	−	+++	+++	++	+++	2	+++	++
30 d	Neocortex	−	−	−	−	−	+++	+++	+++	+++	0	+++	++
30 d	H’campus	−	−	−	−	−	+++	+++	+++	+++	0	+++	++

**Columns 1–5:** The expression of Aβ, APP and hyperphosphorylated tau in neural cells along the flanks of the track was upregulated quickly and transiently. The appearance of oligomeric Aβ in neural cells along the flanks, and along the track (perhaps extracellular) is similarly rapid and transient.

**Columns 6, 7:** The migration of glia to the site and/or proliferation of glia at the site developed more gradually and continued to the 30 d timepoint.

**Columns 8–10:** The death of cells (TUNEL-labelled cells averaged to the whole nearest number) began rapidly, and declined to baseline levels at 19 d. The loss of neuronal nuclei and of synaptophysin labels in axon terminals was detected more slowly, but persisted to 30 d.

**Column 11:** The Perls (Prussian blue) reaction for haem was positive early. Haem-positive deposits increased in prominence to day 14 and persisted to the 30 d time point.

**Column 12:** Fluorescent deposits, shown in several cases to be 4G8^+^ and hyperphosphorylated tau^+^, were detected at 7 d and grew in prominence to 30 d.

Within GFAP^+^ cells, presumably astrocytes, 4G8 labelling was present in the somas and extended along processes (Figures 7C, D). Within the cytoplasm of neurones, the labelling appeared diffuse, and unattached to intracellular organelles. APP labelling was also apparent in the somal cytoplasm of neurones and, in more punctate form, along the GFAP^+^ processes of astrocytes (Figure 7E). This upregulation was detected at 1 d post-lesion, peaked at 3 d and had subsided by 7 d.

Along the flanks of the needle track, the A11 antibody for oligomerised Aβ labelled many small punctate structures, and occasionally larger (up to 10 µm), more complex structures, apparently in the neuropil (Figure 6 B,C). This is consistent with earlier descriptions of oligomerised Aβ forming in synaptic terminals [33], [36], [37]. Larger labelled structures were also observed near human plaques (Figure 6D).

In neocortex, we observed dying (TUNEL^+^) cells only along the needle track (above). As already noted, TUNEL^+^ cells were observed in the flanking regions only in the hippocampus, where the needle penetrated the densely packed pyramidal layer (Figure 5G). Many authors have noted the relative vulnerability of hippocampal neurones to stress in various forms (reviewed [38]).

The increased density of astrocytes at the needle track also extended into the flanking regions (Figure 5B). Nevertheless, micro- and astrogliosis were most prominent at the needle track, suggesting that the gliosis is a response to the neuronal death and haemorrhages caused at that site.

### Time Course of Pathological Changes

#### Summary of neuropathology

Table 2 summarises several features of neuropathology observed. Antigens considered important in age-related dementia (hyperphosphorylated tau, APP, Aβ, oligomeric Aβ) were prominently upregulated in neurones and glia along the flanks of the track for 1–7 d post lesion; this upregulation was transient, fading after 7 d. Gliosis, the migration and/or proliferation of microglia and astrocytes, was detectable at day 3 and increased thereafter, outlasting the upregulation of Aβ-related antigens. Cell death assessed with the TUNEL technique was maximal in the first few days after the lesion, and slowed thereafter. Iron, presumably derived from circulating haemoglobin, was detected along the needle track at 1 d; iron deposits increased in compactness and size to 14 d and remained prominent at 30 d. The formation of ECDs was detected at 7 d and remained prominent at 30 d. In summary, the needlestick lesion induced a prominent but transient upregulation of hyperphosphorylated tau and Aβ-related antigens in cells on the flanks of the track; and several permanent changes (gliosis, neuronal loss, haemorrhage and formation of ECDs) along the track.

#### Western blots

**Figure 8:**
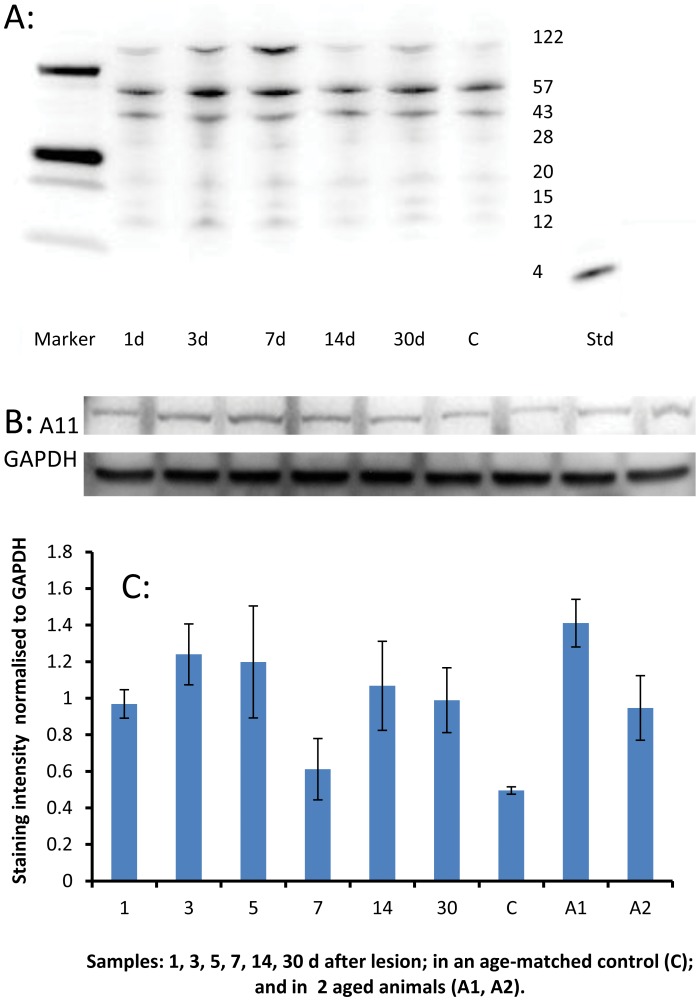
Amyloid-related protein expression in needle stick brain tissue. A: Western blot of tissue near and including a needle track, labelled with the 4G8 antibody (which labels Aβ peptide and precursor molecules such as APP). Protein samples (50 µg/lane) were processed from animals surviving 1 d, 3 d, 5 d, 7 d, 14 d and 30 d after the lesion. Lane C shows a sample from an unoperated (age-matched control) brain. Labelling at 4 kDa (monomeric Aβ) was not obtained. Twin bands were observed at 122 kDa, corresponding to the size of APP. Labelling at molecular weights characteristic of oligomers was obtained (20/15/12 kDa). These bands showed intensification above control (C) levels, most prominently at 3 d and 7 d. As expected, a 4 kDa band was observed for the Aβ_1–40_ standard. B: Western blot of the same tissue, using the A11 antibody (which labels oligomeric Aβ). A11 labelling identified a single band at 100 kDa, presumed to be a pre-fibrillar form of oligomeric Aβ. GAPDH (37 kDa) was used as a loading control. C: Densitometry analysis of the A11 labelling in B, normalised to GAPDH. Labelling (for oligomeric Aβ) shows limited upregulation at 3 d and 5 d, and again in the older animals (A1 and A2). Error bars represent standard error of the mean, n = 4 separate experiments.

A Western blot using the 4G8 antibody, which labels Aβ and its precursors, confirmed one element of the time course shown in Table 2. In Figure 8A, 4G8 labelling of species at 20 kDa, 15 kDa and 12 kDa represent oligomers of monomeric Aβ (4 kDa). These bands reflect an upregulation (above control) at 1 d, 3 d (peak) and 7 d and then a return towards control levels. Twin bands were detected at 122 kDa, corresponding to the size of APP. A comparable blot for oligomeric Aβ, with the A11 antibody (Figure 8B), also showed upregulation of a 100 kDa immunoreactive species at 1–3 d, but persisting longer, to 30 d (Figure 8C).

### Comparison with Human Senile Plaques

**Figure 9:**
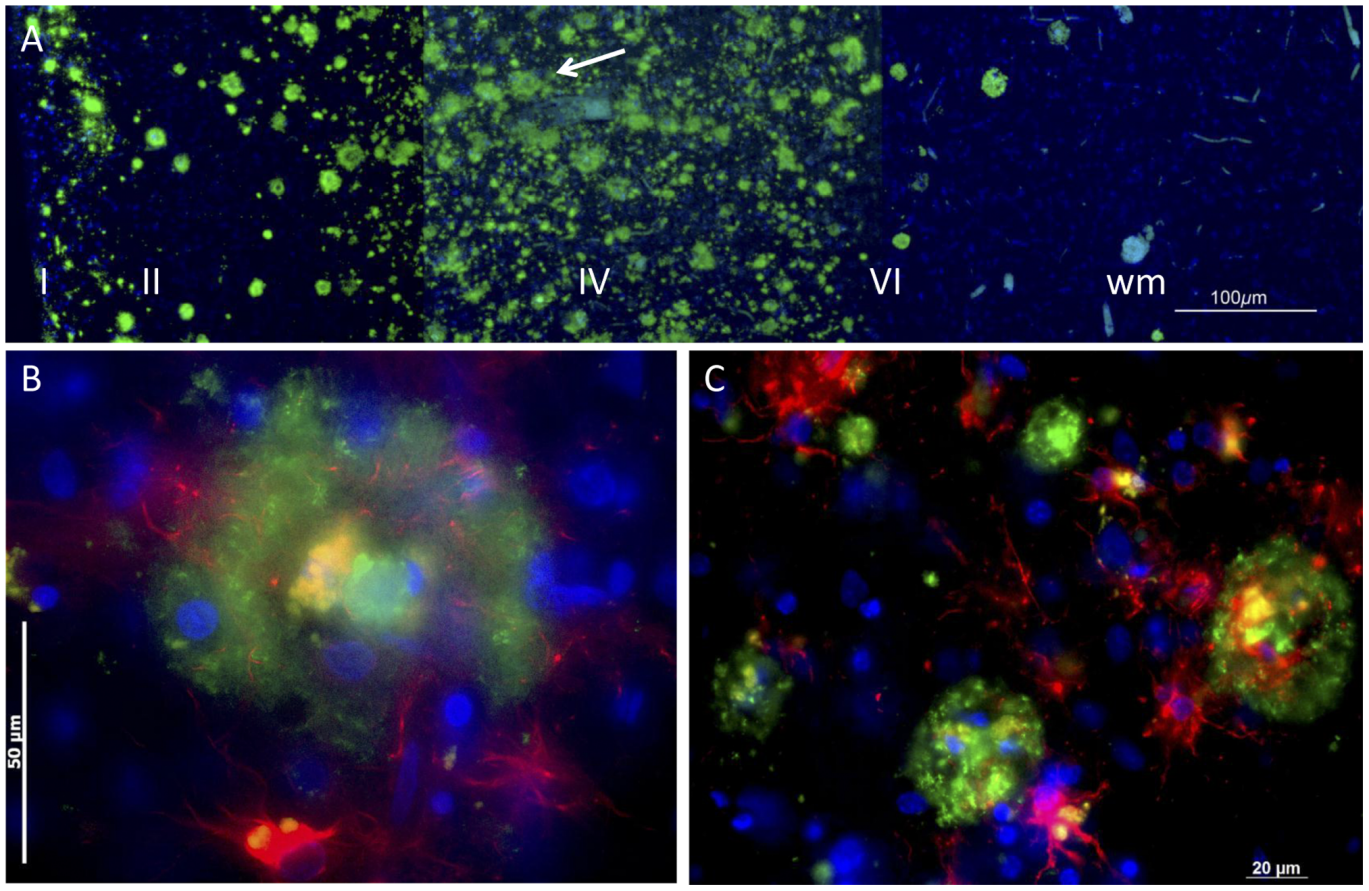
Concentric organisation of human plaques. A: At low power the long-established pattern of plaques characteristic of senile dementia is demonstrated with the 4G8 antibody. The arrow points to a series of plaques arranged in a line, along a blood vessel. B: At higher power, many plaques show a concentric arrangement. The core is cellular, the cells often containing autofluorescent material, including lipofuscin and hemosiderin. This core is surrounded by a shell of 4G8-labelled Aβ (green), with astrocytes (GFAP-labelled, red) within and outside the cell. The blue fluorescence is bisbenzimide labelling of nuclear DNA. C: The concentric arrangement was a common feature of plaques.

Figure 9A shows plaques in the neocortex (temporal lobe) of an AD case, labelled with the 4G8 antibody. Plaques are present in all layers of the cortex, being relatively sparse in layer II; they are also present, but sparse, in the white matter, confirming earlier studies [5]. Many are rounded in shape; several large plaques are aligned along a relatively large vessel in layer IV-V (arrow). The largest are ∼50 µm in diameter.

**Figure 10:**
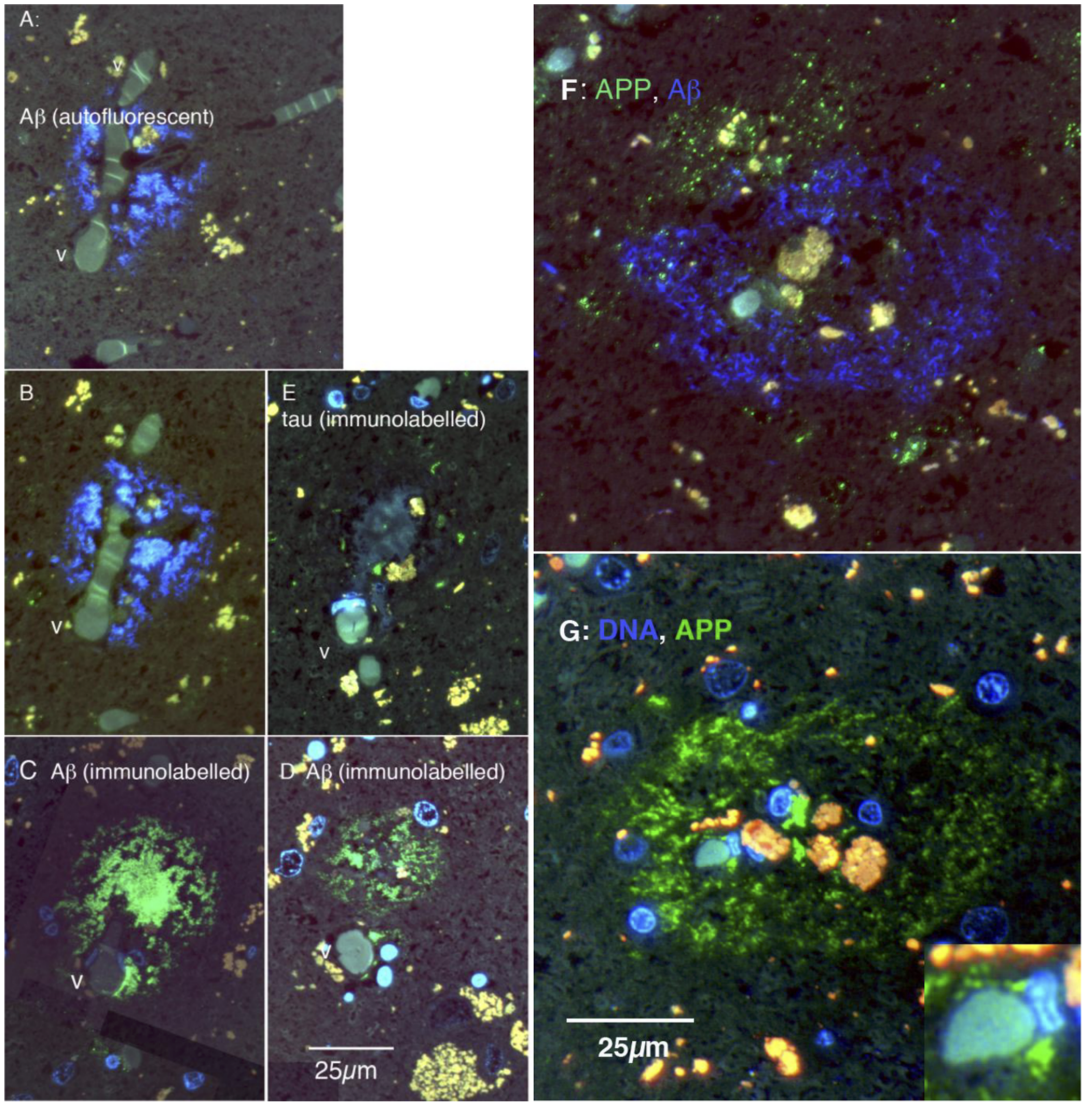
Cellular organisation of human plaques, seen in serial, thin (0.1 µm) sections. A–E are sections of one plaque, F and G of another. A: The blue fluorescence is autofluorescence from amyloid in the plaque. It surrounds a branch point of a capillary-size vessel (v). B: 0.8 µm away, the amyloid autofluorescence is still prominent. C: 1.0 µm from A (and 0.2 µm from B) the plaque is labelled green with the 4G8 antibody. D: 9 µm from A (in the same direction), the plaque remains clearly labelled with the 4G8 antibody. E: 4 µm from A, the same vessel (v) is apparent; small elements of tau labelling (green) are shown, in the area of the plaque. F: Blue autofluorescence shows amyloid forming a shell surrounding red and yellow autofluorescent bodies. Labelling for APP (green) shows punctate APP^+^ elements forming an incomplete shell outside the amyloid shell. G: The blue fluorescence in this panel is bisbenzimide labelling for nuclear DNA. 4G8 labelling (green) shows Aβ forming a shell around a cellular core, which includes the vessel, some nuclei and autofluorescent material. The inset shows a vessel at the centre of the plaque, at higher power.

Viewed with a DNA label included (Figure 9B), plaques appeared highly cellular, containing nuclei with normal chromatin and TUNEL^−^ (data not shown). As previous reports have noted, many of the cell nuclei found in plaques are of glial cells; Figure 9B shows that astrocytes (immunolabelled red) are present in and around the plaque. Endothelial cells of blood vessels are also found within the 4G8^+^ regions of plaques (Figure 10). These nuclei do not appear pyknotic or degenerative, suggesting that the environment is not cytotoxic.

Plaques show a strong concentric organisation. Figures 9B and 10 F,G show this concentric organisation for a single plaque; it was commonly observed (Figure 9C). The APP/Aβ^−^ (4G8^−^) labelled material seems to form a shell, inside which some nuclei and autofluorescent bodies, perhaps lipofuscins and/or haemosiderin, are found. In favorable preparations, evidence could be seen of an incomplete shell of APP (Figure 10F) or GFAP^+^ (astrocytic) structures (Figure 9B) external to the shell of Aβ.

Confirming previous studies [4], [9], we noted that many plaques formed in close relationship to small blood vessels. In Figure 10A–E, the five panels are from adjacent sections through the same plaque, which has formed around the branch point of capillary-sized vessels. Figures 10A and 10B show the plaque by autofluorescence (blue autofluorescence of plaques has been described previously and attributed to Aβ [27], [28]); Figures 10C and 10D show the same plaque immunolabelled with 4G8, in sections 8 µm away; and Figure 10E shows that small elements of tau are present in the plaque. Figures 10F,G show adjacent sections of another plaque from the same case. In F, the plaque is autofluorescent and APP is labelled red, by immunohistochemistry. Panel G shows the same plaque immunolabelled with 4G8, and demonstrates a small capillary-sized vessel, with an endothelial nucleus adjacent (inset), inside the 4G8^+^ shell. Not every section through a plaque showed a blood vessel, but a close relationship was common. A statistical analysis of the spatial relationship of plaques to vessels is reported in [4], [5].

A key point in Cullen and colleagues’ [4], [5] conclusion/hypothesis that plaques form at the site of microhaemorrhages was evidence that the plaque-ridden brain is ridden also with small deposits of haem, detected with the Prussian blue/Perls reaction, which co-localise with plaques. We searched for this co-localisation using the Prussian blue reaction to detect iron and amyloid-binding dyes. As noted by Cullen and colleagues [5], it is difficult to test this co-localisation using immunohistochemistry, because the high acidity of the Prussian blue reaction destroys antigens.

**Figure 11:**
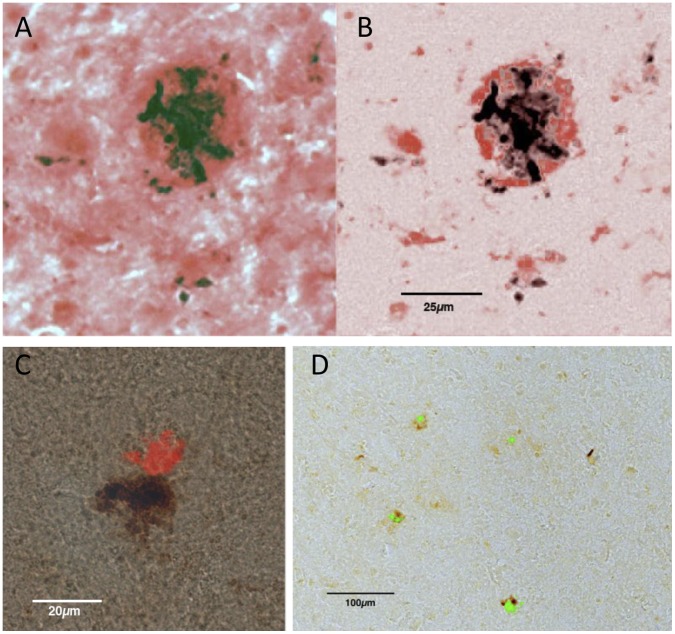
Co-localisation of haem and amyloid labelling in human neocortex. A,B: The dark material is haem, identified with the Perls (Prussian blue) reaction, with DAB as the reporter molecule. The red labelling is Congo red, which labels amyloid. In the right hand panel the background Congo red labelling has been reduced. The largest haem deposit is surrounded by a shell of Congo red. Smaller haem deposits co-localise side-by-side with Congo red. C: Side-by-side co-localislation of haem and Congo red, in another plaque. D: Co-localisation of haem and Thioflavin S (green) in several plaques.

Figure 11A shows a human plaque labelled for Aβ with Congo Red, and for haem using the Prussian blue reaction, with DAB as the reporter reaction. When the peaks of Congo red labelling were isolated (Figure 11B) it was apparent that the major site of Prussian blue labelling was surrounded with a ring of Congo red; and that several smaller Prussian blue granules were also related to Congo red labelling. Figure 11C shows another example of the close apposition of haem and amyloid, in a plaque, using Congo red. Figure 11D shows the same relationship for a group of plaques, this time with the amyloid labelled green with Thioflavin S, again selecting the peaks of dye labelling.

## Discussion

The present experiments show that a penetrating injury of the cerebral cortex creates distinct neuropathologies along the track of penetration, and in the tissue that flanks the track. Along the track, there is mechanical disruption of tissue, loss of neurones and synapses, microglial proliferation/invasion and the extracellular formation of haem^+^, hyperphosphorylated tau^+^ and APP/Aβ^+^ deposits, all long-lasting changes. In the flanking regions, which can be compared with the penumbra of ischaemic tissue which surrounds an intracerebral haemorrhage [39], there is little or no loss of neurones (except in the pyramidal layer of the hippocampus); the most prominent changes are the upregulation of expression of APP/Aβ and hyperphosphorylated tau; and these upregulations are intracellular and transient.

### Implications

The changes that occur along the track resemble the plaques and neuritic tangles found in the ageing human brain, associated with dementia. In particular, the extracellular deposits which formed along the track were similar in size (up to 50 µm), were surrounded and invaded by microglia and macroglia (astrocytes), were labelled by antibodies (4G8, 6E10 and AT8) for APP, Aβ and hyperphosphorylated tau, and contained and were surrounded by oligomerised Aβ. Further, extracellular deposits of haem^+^ material also formed along the track, suggesting co-localisation of haem and Aβ^+^ deposits, as reported for human senile plaques [4], [5] and confirmed in Figure 9.

The present results allowed as a test of the prediction, arising from a specific hypothesis of senile plaque formation [5], [8], that haemorrhage can induce the formation of plaque-like deposits in the otherwise healthy brain. The co-occurrence of deposits of haem and APP/Aβ^+^ deposits, both narrowly confined to the penetration track (Figures 1, 2), provides support for this prediction, although it does not, of course, prove causation.

It can be noted, however, that where there is no haemorrhage, plaque-like deposits do not form. In the present experiments, for example, the upregulation of APP and Aβ in tissue on the flanks of the penetration track is an established marker of brain trauma [40], [41], [42], [43]. The flanks responded to the penetration, but haemorrhage did not extend to the flanks, and extracellular deposits did not form there. Several earlier reports also support this negative correlation (no haemorrhage, no plaque-like structures). For example, non-haemorrhagic ischemia induces brain damage and cognitive loss comparable to human dementia, without the formation of plaques [14], [15], [44]. Correspondingly, in a model of ischaemic stroke in the Tg2576 mouse (in which the transgene causes plaque formation [45]), ischaemia without haemorrhage did not increase the formation of Aβ^+^ plaques. In humans, most cases of stroke are non-haemorrhagic [46], and plaques are not associated with the resultant brain damage and dementia.

In their review of the neuropathology of human stroke, Hossmann and Heiss [46] write that most spontaneous intra-cerebral haemorrhages occur in deep hemispheric structures (so not in the cortex) and in small arteries. If it proves correct that plaques form at sites of bleeding from cortical capillaries, as argued by [5], [8], these conclusions may need to be updated. That is, intracerebral haemorrhage may be much more common than indicated by the aetiology of stroke; may be much more common in the cerebral cortex than in deeper structures; and may occur most frequently in capillaries, rather than small arteries.

### Relationship to Previous Work–Brain Injury, Haemorrhage, Aβ, Tau and Plaques

The present evidence that a form of traumatic brain injury (TBI, in this case a needle stick) causes haemorrhage, the co-localised formation of plaque-like deposits and increased expression of Aβ and tau in surrounding tissues, could have been anticipated from prior studies.

In humans, closed-head TBI (typically a blow to the head) has been reported to cause small-vessel haemorrhages (petechial bleeding) [47], [48], and to be associated with the deposition of Aβ and/or the formation of plaques [40], [41], [42], [43], [49], [50], [51], [52], [53], hyperphosphorylated tau [35], [54] and dementia [55], [56], [57]. In the rat, needle stick injury has been reported previously (confirmed in Figure 7) to induce upregulation of APP and related enzymes in surrounding tissue [29], [58]; the present observation that 4G8 and AT8 labelling is upregulated appears to be novel. In Tg2576 mice, repeated mild, closed-skull trauma caused the formation of Aβ^+^ plaque-like deposits [59]. These relationships have been reported largely separately; this study links them in an experimental test of the idea that trauma-induced haemorrhage can induce the formation of plaques.

Other workers have proposed other links from TBI to plaque deposition; for example that plaque formation is mediated by damage to tracts of nerve fibres in which trauma has induced an upregulation of Aβ [51]. The present study does contribute to defining the link between injury and plaque formation, but previous studies [60], [61] have proposed a link in the binding of Aβ to haemoglobin, and the induction of oligomerisation of Aβ which results. As previously argued [8], this binding could be a step from haemorrhage to plaque formation.

### Plaques and ECDs–toxic Foci or Scars?

The question whether senile plaques are quiescent scars or foci of toxicity bears on the mechanism, and therefore on the treatment, of age-related dementia. The idea that the senile plaque is an active site of toxicity arises from extensive evidence of the toxicity of various forms of Aβ (monomeric, oligomeric and polymeric/insoluble), summarised in [62]. The evidence includes studies of the interaction of Aβ with other abnormal proteins found in association with plaques, and studies of the interaction of various metals with Aβ. This evidence formed the basis for clinical trials in which the amyloid ‘load’ of the brain was reduced, in the hope of reducing toxicity. The trials have been disappointing, however, though the search for an amyloid-related treatment continues (for a sympathetic, accessible review, see [63]).

The evidence that the senile plaque is a non-toxic scar, formed at the site of an ‘upstream’ pathology such as a small-vessel bleed, includes evidence that plaques form around blood vessels and contain blood specific molecules (haem, clotting factors, even red cells) [4], [5], [9]; and evidence (reviewed in [8]) that ‘Alzheimer-like’ (i.e. plaques and tangles) and cerebrovascular pathologies co-occur in many cases studied post-mortem, that the risk factors for age-related dementia and for cardiovascular disease overlap extensively, and that oxidative stress and hypoxia are prominent in the dementing brain. Other authors [64], [65] have developed the additional argument, that plaques may be a defensive response to the hypoxia, oxidative stress and iron accumulation stress prominent in the brain undergoing dementia.

### The Flanking Region–a Region of Death or Survival?

Neuronal death could not, in our data, be detected in the region flanking the needle track, in which the expression of APP, Aβ and tau was upregulated transiently by the penetrating injury; Aβ expression in these conditions appears not to be toxic. Correspondingly, mature human plaques appear cellular and organised, with a population of active glial cells, confirming previous reports [66]. That is, the regions surrounding penetrating injury or plaques do not appear to be regions of increased cell death.

What then might be the effect of the transient upregulation of APP and Aβ induced in our needle stick model, up to 500 µm from the needle track? One clue comes from the observation of the effect of needle stick penetration of the retina [67], [68]. These workers tested the neuroprotective effects of growth factors injected into the vitreous humour on photoreceptor death in rat models in which a genetic mutation or light damage caused premature photoreceptor degeneration. Injections of fibroblast growth factor and ciliary neurotrophic factor (CNTF) slowed the degeneration; and sham injections which penetrated the retina also induced a slowing of photoreceptor death over a wide region of retina, and induced the upregulation of brain derived neurotrophic factor and CNTF. We are currently testing whether a penetrating wound of the brain produces a similar ‘halo of invulnerability’, and whether that halo corresponds spatially and temporally with the upregulation of APP, Aβ, tau and other candidate peptides and cytokines.

### Limits

Several limitations of the present observations deserve note. First, the penetrating injury model does not separate the mechanical impact of the needle from the haemorrhage which occurs along the track. Causal connection between haemorrhage and the formation of plaque-like deposits has to be argued from additional grounds (above).

Second, the 4G8 and 6E10 antibodies are established labels for Aβ, but also label the precursor protein APP. Present observations that labelling with these antibodies increases strongly but transiently in tissue around the needle track indicates that Aβ expression, and perhaps its secretion by cells (neurons and glia) near the track, are upregulated. Re-analysis with monomer-specific antibodies is required to establish whether the cells generate monomeric Aβ.

Third, we observed that, in the rat brain, plaque-like ECDs did not stain with Congo red or Thioflavin S, suggesting that they do not have the same β-sheet structure as senile plaques in the human brain. We could not therefore demonstrate the co-localisation of haemorrhage-related iron with Congo red or Thioflavin S, as was possible for human plaques. Nevertheless, both the Perls reaction product and 4G8^+^ or 6E10^+^ extracellular deposits (plaques) were tightly confined to the needle track, suggesting that the ECDs form where the needle causes haemorrhage.

Fourth, because there are no histochemical stains for haem, we used Perls staining for iron as a surrogate marker. While it is possible that some of the iron observed may originate from cell damage rather than haemorrhage, Perls staining has been extensively used by neuropathologists to assess intracerebral bleeding [69], [70] and the close proximity of iron deposition to blood vessels gives us confidence that this is an appropriate marker of haemorrhage.

### Vasculopathy vs Proteopathy

Finally, comment is appropriate on the logical relationship between apparently competing ideas of the pathogenesis of Alzheimer’s dementia, which suggest that the dementia is due on the one hand to cerebrovascular disease [4], [8], [9], [15] and, conversely, that the condition is a proteopathy, caused by the formation of toxic forms of proteins [71], [72].

At first glance, the two proposals appear mutually exclusive; but haemorrhage induces hypoxia and hypoxia upregulates Aβ expression, and may induce an amyloid proteopathy. De la Torre’s emphasis [44] on ischaemia caused by capillary hypofunction seems distinct from the haemorrhage hypothesis tested here, but if cerebral ischaemia can induce haemorrhage [73], the two ideas converge. We would still emphasise that only the capillary haemorrhage idea can account for the distribution, size and features of plaques [8], but a fair reading of the evidence does not, we suggest, lead to the exclusion of any of these ideas. What is at issue is the pathogenesis of plaques, and whether it begins with vascular breakdown.
